# *In vitro* Production of IL-6 and IFN-γ is Influenced by Dietary Variables and Predicts Upper Respiratory Tract Infection Incidence and Severity Respectively in Young Adults

**DOI:** 10.3389/fimmu.2015.00094

**Published:** 2015-03-04

**Authors:** Huicui Meng, Yujin Lee, Zhaoyong Ba, Jennifer A. Fleming, Emily J. Furumoto, Robert F. Roberts, Penny M. Kris-Etherton, Connie J. Rogers

**Affiliations:** ^1^Department of Nutritional Sciences, Pennsylvania State University, University Park, PA, USA; ^2^Department of Food Science, Pennsylvania State University, University Park, PA, USA

**Keywords:** activation markers, T-cell proliferation, cytokine secretion, cold or flu incidence and severity, host factors

## Abstract

Assessment of immune responses in healthy adults following dietary or lifestyle interventions is challenging due to significant inter-individual variability. Thus, gaining a better understanding of host factors that contribute to the heterogeneity in immunity is necessary. To address this question, healthy adults [*n* = 36, 18–40 years old, body mass index (BMI) 20–35 kg/m^2^] were recruited. Dietary intake was obtained via 3-day dietary recall records, physical activity level was evaluated using the International Physical Activity Questionnaire, and peripheral blood mononuclear cells were isolated from peripheral blood. Expression of activation markers on unstimulated immune subsets was assessed by flow cytometry. T-cell proliferation and cytokine secretion was assessed following *in vitro* stimulation with anti-CD3 or lipopolysaccharide. Furthermore, the incidence and severity of cold or flu symptoms were obtained from self-reported upper respiratory tract infection (URTI) questionnaires. The relationship between activation marker expression on T cells and T-cell effector functions; and *in vitro* cytokine secretion and URTI was determined by linear or logistic regression. CD69 and CD25 expression on unstimulated T cells was significantly associated with T-cell proliferation and interleukin-2 secretion. Incidence and severity of cold or flu symptoms was significantly associated with *in vitro* interleukin-6 and interferon-gamma secretion, respectively. Furthermore, host factors (e.g., age, BMI, physical activity, and diet) contributed significantly to the relationship between activation marker expression and T-cell effector function, and cytokine secretion and cold and flu status. In conclusion, these results suggest that lifestyle and dietary factors are important variables that contribute to immune responses and should be included in human clinical trials that assess immune endpoints.

## Introduction

Host immune function is influenced by endogenous factors (e.g., age, gender, genetics), as well as various exogenous factors (e.g., diet, physical activity, alcohol consumption) (1–6). Thus, heterogeneity in both innate and adaptive immune responses exists even among healthy adults. This inter-subject variability makes it challenging to evaluate the effect of dietary or lifestyle interventions on immune function as many of these interventions have moderate effects as compared to pharmacological intervention trials (5, 7). Additionally, cost and feasibility often limit the sample size in clinical trials. Thus, gaining a better understanding of host-related factors that may contribute to the variability in innate and adaptive immune responses in healthy adults is necessary to adequately control for the contribution of these influences in clinical trials that assess immunity.

The assessment of innate and adaptive immune function often involves complex assay methodologies; thus large clinical trials frequently are not designed to quantify these types of immunologic outcomes. Many human clinical trials measure serum makers (e.g., antibody titers, cytokine levels) as indicators of immune function because of ease of collection, assessment, and storage (5). However, these endpoints alone may not adequately capture the immune response. Thus, additional studies are needed to assess functional immune outcomes (e.g., T-cell proliferation, *in vitro* cytokine secretion) concurrently with the phenotypic characterization of immune cell populations via flow cytometry to determine if cell surface marker expression on immune cells can serve as a biomarker for effector function (e.g., proliferation and cytokine secretion).

Numerous human clinical trials have evaluated the effect of lifestyle interventions on the incidence and severity of cold or flu symptoms captured in self-reported upper respiratory tract infection (URTI) questionnaire data. The immune response of the host is known to be an important component of the pathogenesis of cold or flu infection (8). However, very few studies have quantified immune function (inflammatory cytokine responses or T-cell function) concurrently with the self-reported URTI questionnaire data to determine if any immune outcomes are correlated with cold or flu symptomology. Two studies have reported an increase in salivary IgA concentration in subjects who had a lower incidence and severity of URTI symptoms (9, 10). In a third study, the risk of URTI in athletes was associated with antigen-stimulated interleukin (IL)-10 production and salivary IgA secretion (11). All three studies demonstrate that changes in immune function can be correlated with reduced symptoms of URTI. However, the goal of these studies was to determine if exercise reduced URTI, so the relationship between URTI symptomology, salivary IgA, and IL-10 may be confounded by the exercise intervention. To date, no studies have examined inflammatory cytokine response or T-cell effector function in subjects who completed self-reported URTI questionnaire data to determine if T-cell effector function or inflammatory cytokine production was related to URTI incidence or severity.

Therefore, the goals of the current study were (1) to determine which endogenous and exogenous host factors contribute to the heterogeneity in innate and adaptive immune responses among healthy subjects; (2) to determine if activation marker expression on freshly isolated T cells, macrophages, or dendritic cells (DCs) is associated with functional outcomes, i.e., anti-CD3-induced T-cell proliferation and cytokine [IL-2 and interferon-gamma (IFN-γ)] secretion or lipopolysaccharides (LPS)-stimulated cytokine [tumor necrosis factor-alpha (TNF-α) and IL-6] secretion from peripheral blood mononuclear cells (PBMCs), respectively; and (3) to determine if T-cell proliferation and/or *in vitro* inflammatory cytokine production is associated with self-reported incidence and severity of cold or flu symptoms collected using a validated URTI questionnaire.

## Materials and Methods

### Participants

Healthy subjects (*n* = 36, 25 women and 11 men) 18–40 years of age were recruited for the study. Exclusion criteria included: body mass index (BMI) greater than 40 kg/m^2^, smoking and/or use of other tobacco products, blood pressure ≥140/90 mm Hg, use of blood pressure- or cholesterol-lowering medications, history of myocardial infarction, stroke, diabetes mellitus, liver disease, kidney disease and thyroid disease (unless controlled by medication and blood results within the previous 6 months were provided), lactation, pregnancy or desire to become pregnant during the study, clinical diagnosis of inflammatory bowel disease (e.g., Crohn’s disease or ulcerative colitis), excessive alcohol consumption (>14 standard drinks/week), chronic use of anti-inflammatory medications (unless able to discontinue), and refusal to agree to give blood or plasma for the length of the study. A complete blood count and standard biochemistry panel was obtained at screening to rule out the presence of illness (autoimmune disease, cancer, and immunodeficiency). Blood pressure was measured according to the Joint National Committee 7 Guidelines (12).

### Recruitment and screening

Participants were recruited through advertisements in the local newspaper and university e-mail lists. Potential participants who called or emailed to indicate interest in participating in the study were given information about the study, and if still interested, were contacted and screened using a series of medical and lifestyle questions. Qualified participants were scheduled for clinic screening at the Penn State Clinical Research Center (CRC). After written informed consent was provided, participants’ height, weight, waist circumference, and blood pressure were measured, followed by a fasting blood draw for a complete blood count and metabolic and immunologic endpoints. BMI was calculated according to body weight and height measured. From the participants who were screened, 36 were eligible to participant in the study. All the experiments in this study were performed with approval of the Institutional Review Board of the Pennsylvania State University-University Park campus (University Park, PA, USA).

### Diet assessment

Dietary intake of the participants was obtained via 24 h dietary recalls for three continuous days. Briefly, participants were asked to recall their intake of food and beverages during breakfast, lunch, dinner, and snacks in the three continuous days according to detailed instructions provided by trained staff. Portion size of each food item was also provided. Daily intake of total calories, macronutrients, vitamins, minerals, caffeine, and alcohol was analyzed based on the recorded food intake of participants using Food Processor SQL software (ESHA Research, Salem, OR, USA).

### Physical activity assessment

Physical activity level of participants was evaluated using the International Physical Activity Questionnaires (IPAQ) as previously reported (13). Briefly, the participants recorded the activities they performed during each 15-min interval for 3 days, including one weekend day. The activities were categorized from one to nine depending on their intensity as previously described (13). Daily intensity of physical activity was calculated by averaging the approximate metabolic equivalent of tasks (METs) of physical activities (categories three to nine) performed over a 24-h period (96 periods of 15 min).

### Upper respiratory tract infection questionnaire

Participants (*n* = 34) completed a self-administered URTI questionnaire, which was developed from established, frequently used instruments on the incidence (whether or not have experienced colds or flu episodes, with symptoms including a sore throat, runny or stuffy nose, coughing sneezing, fever, headache, general aches and pains, fatigue, and discomfort) and severity (total number of days with cold or flu symptoms) of cold or flu symptoms over the past month. Participants were instructed to recall the occurrence of cold or flu in the last month, and advised on questionnaire completion. Two participants did not provide any information in their URTI questionnaires for unknown reasons.

### Blood sample collection and immunological assays

Blood (50 ml) was collected in sterile EDTA (K2)-coated blood tubes (BD Biosciences, San Jose, CA, USA) after a 12-h fast by trained staff in CRC of Pennsylvania State University.

#### Serum markers

Total cholesterol (TC) and triglycerides (TG) were measured by enzymatic procedures (Quest Diagnostics, Pittsburgh, PA, USA; coefficient of variation CV <2% for both). High-density lipoprotein (HDL) cholesterol was estimated according to the modified heparin–manganese procedure (CV <2%). The Friedewald equation was used to calculate low-density lipoprotein (LDL) cholesterol = TC − (HDL cholesterol + TG/5) (14). Insulin was measured by radioimmunoassay (Quest Diagnostics). Glucose was determined by an immobilized enzyme biosensor using the YSI 2300 STAT Plus Glucose and Lactate Analyzer (Yellow Springs Instruments). Serum high-sensitivity C-reactive protein (hs-CRP) was measured by latex-enhanced immunonephelometry (Quest Diagnostics; assay CV <8%).

#### Isolation of immune cells

Human blood was diluted 1:2 with phosphate buffer saline (PBS; Mediatech, Manassas, VA, USA), gently layered on top of lymphocyte separation media (LSM; Corning, Manassas, VA, USA), and centrifuged at 1600 rpm with low speed and no brake for 30 min at room temperature. PBMCs were collected at the plasma/LSM interface; washed twice with complete media RPMI 1640 (Mediatech) containing 10 mM HEPES (Mediatech), 10% heat-inactivated fetal bovine serum (Gemini, West Sacramento, CA, USA), 2 mM L-glutamine (Mediatech), 0.1 mM non-essential amino acids (Mediatech), 1 mM sodium pyruvate (Mediatech), 100 U/ml penicillin/streptomycin (Mediatech), and 55 μM 2-mercaptoethanol (Life Technologies, Grand Island, NY, USA) at room temperature; and counted for use in functional and phenotypic analyses.

#### Lymphocyte proliferation assay

Peripheral blood mononuclear cells (2 × 10^6^/ml) were incubated with 0 or 1 μg/ml plate-bound purified mouse anti-human CD3 antibody (Life Technologies) in flat-bottomed 96-well plates. After 54 h in culture, the cells were pulsed with [^3^H] thymidine (1 μCi/well; Perkin-Elmer, Waltham, MA, USA) and harvested 18 h later. Following incubation, cells were harvested onto glass fiber filter mats (Perkin-Elmer) via a MicroBeta FilterMate-96 Harvester (Perkin-Elmer). Incorporated radioactivity was measured by liquid scintillation counting on a 2450 MicroBeta plate counter (Perkin-Elmer). Each assay was performed in triplicate. The proliferative response was expressed as a stimulation index (SI) calculated by dividing the mean counts per minute (cpm) of anti-CD3-stimulated T cells by the mean cpm of unstimulated (media only) cells.

#### Cytokine secretion assays

Peripheral blood mononuclear cells (2 × 10^6^/ml) were stimulated with 1 μg/ml plate-bound purified mouse anti-human CD3 antibody (Life Technologies), or 10 μg/ml LPS (Sigma-Aldrich) in flat-bottomed 96-well plates. Supernatants from LPS plates were harvested and frozen after a 4-h incubation, and supernatants from anti-CD3 plates were harvested and frozen after 48 h. IFN-γ and IL-2 secretion from anti-CD3-stimulated PBMCs, and TNF-α and IL-6 secretion from LPS-stimulated PBMCs were measured using the Human ELISA MAX™ Deluxe (BioLegend, San Diego, CA, USA) as per manufacturer instructions. Each assay was performed in triplicate.

#### Flow cytometric analyses

Peripheral blood mononuclear cells were washed twice in PBS at 4°C. Fc receptors on PBMCs were blocked by incubation with 1 μg purified mouse anti-human CD16 (BioLegend)/1 × 10^6^ cells for 15 min at 4°C. PBMCs were stained with fluorescence-labeled antibodies (1 μg/1 × 10^6^ cells) to the following cell surface markers: CD3e, CD4, CD8a, CD69, CD25, CD11c, CD14, HLA-DR. Antibody isotype controls included: mouse IgG_2a_ and mouse IgM. All antibodies except CD16 were purchased from BD Biosciences. Following incubation with the conjugated antibodies for 30 min at 4°C, cells were washed twice in PBS and then fixed in cytofix (BD Biosciences) for flow cytometric analyses. Lymphoid and myeloid cells were gated on forward vs. side scatter and a total of 25,000 events were analyzed on a FC500 Benchtop Cytometer (Beckman Coulter, Pasadena, CA, USA). Flow cytometric analyses were plotted and analyzed using FlowJo 7.6 (Tree Star, Ashland, OR, USA).

### Statistical analyses

Spearman’s rank correlation was used to determine the relationship between IL-2 and IFN-γ secretion from T cells and T-cell proliferation. Differences in IFN-γ secretion from anti-CD3-stimulated T cells between participants with and without self-reported cold or flu episodes in the past month were determined using unpaired *t*-test. Differences in IL-6 secretion from LPS-stimulated PBMCs between participants with and without self-reported cold or flu episodes in the past month were determined using Wilcoxon–Mann–Whitney test. Correlations between LPS-induced IL-6 secretion and anti-CD3-induced IFN-γ secretion and total number of days with cold or flu symptoms was determined via Spearman’s rank correlation.

For all linear, logistic, and Poisson regression models, potential confounders (listed in Tables 3 and 4) were selected based on previous published reports demonstrating a relationship between the variable of interest (e.g., BMI, vitamin D, zinc, etc.) and immune outcomes (2, 3, 6, 15–31). Confounding variables varied in different models, and were selected in each model based on their ability to change the slope of the regression line for the predictor. Briefly, a full model was fitted with either effector function or incidence and severity of cold or flu infection as the dependent variable and activation marker expression and all potential confounding factors mentioned above as the independent variables (predictors). Then, the potential confounding factor showing the smallest contribution to the model was removed, and the percent change of β for predictor in the current model relative to β in the full model was calculated. If the change was within ±10%, this potential confounding factor was deleted from the model. The potential confounding factors were removed from the model one by one based on the 10% change criteria until all the variables remaining in the model were significant at *p* ≤ 0.05 level or the percent change of β was beyond ±10% range. Automated backward and stepwise elimination were also performed to determine which variables remained in the final model. Similar findings were obtained using all three model-building techniques.

Statistical significance was accepted at the *p* ≤ 0.05 level. All data were analyzed using Statistical Analysis System (SAS, Version 9.4, Cary, NC, USA). Graphs were plotted using GraphPad Prism 5 (La Jolla, CA, USA).

## Results

### Participant characteristics

Anthropometric measurements, blood pressure, biochemical characteristics, physical activity, and dietary intake of participants are presented in Table 1. All 36 participants (25 females and 11 males) completed the study. The participants were healthy, young adults (mean age of 28.3 ± 1.0 years). The average BMI was 24.0 ± 0.4 kg/m^2^; 26 (72.2%) participants were normal weight, 9 (25.0%) were overweight, and 1 (2.8%) was obese. Their blood pressure was normal and waist circumference, fasting blood glucose, insulin, lipids and lipoproteins, and CRP levels were within the normal range (Table 1). Dietary intake and physical activity was assessed from self-reported 3-day dietary recall records and IPAQ responses, respectively. The median daily physical activity intensity (based on self-reported responses) was estimated to be 3.1 METs (range 2.4–5.2 METs). The average daily total calorie intake of participants calculated from 3-day dietary recall records was estimated to be 2281.0 ± 130.8 kcal. The daily intake of macronutrients, vitamins, minerals and n-3 polyunsaturated fatty acids (n-3 PUFA), caffeine, and alcohol are reported in Table 1.

**Table 1:** **Demographic characteristics of participants^a^**.

Characteristics	Values (*n* = 36)
Age (year)	28.3 ± 1.0
Male, *n* (%)	11 (30.6%)
Body mass index (kg/m^2^)	24.0 ± 0.4
≤24.9	26 (72.2%)
25.0–29.9	9 (25.0%)
≥30	1 (2.78%)
Waist circumference (cm)	85.2 ± 1.3
Blood pressure (mm Hg)
Systolic	106.3 ± 1.6
Diastolic	72.6 ± 1.1
Glucose (mg/dl)	87.4 ± 1.2
Insulin (mg/dl)	5.2 ± 0.7
CRP (mg/l)	2.5 ± 0.9
Lipids and lipoproteins (mg/dl)
Total cholesterol (TC)	164.7 ± 4.8
LDL cholesterol	92.6 ± 4.3
HDL cholesterol	54.9 ± 1.9
Triglyceride (TG)	85.8 ± 4.8
Physical activity intensity (METs/d)^b^	3.1 (2.4–5.2)
Dietary intake^b^ of
Total calories (kcal/day)	2281.0 ± 130.8
Carbohydrate (g/day)	281.1 ± 17.7
Protein (g/day)	88.4 ± 6.1
Fat (g/day)	90.2 ± 6.0
Vitamin C (mg/day)	69.5 ± 10.7
Vitamin D (IU/day)	106.1 ± 29.3
Vitamin E (mg/day)	3.1 ± 0.5
Iron (mg/day)	14.3 ± 1.2
Selenium (μg/day)	40.2 ± 4.4
Zinc (mg/day)	6.0 ± 0.6
n-3 PUFA (g/day)	0.6 ± 0.1
Caffeine (mg/day)	71.3 ± 14.6
Alcohol consumption (g/day)	2.2 ± 0.9

*^a^Values are presented as mean ± SEM, or median (range) or *n* (%) depending on the variable*.

*^b^Physical activity and dietary intake were assessed from self-reported responses to IPAQ and 3-day dietary recall records, respectively*.

### T-cell proliferation and cytokine secretion

**Figure 1:**
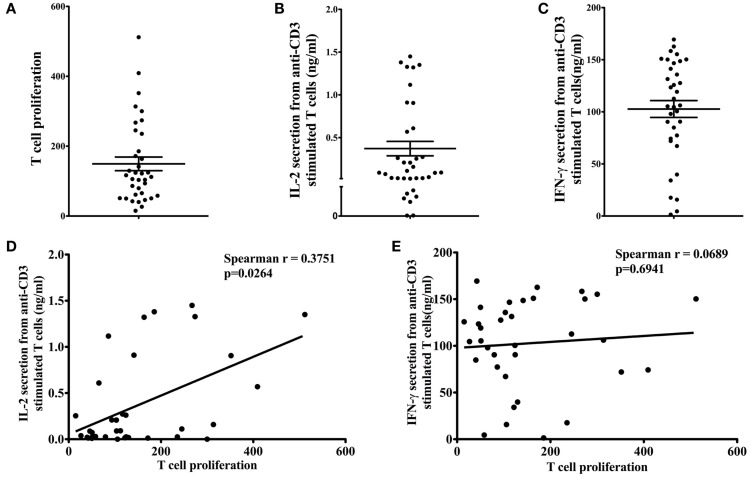
**Effector function of T cells**. Proliferation of T cells **(A)** in response to anti-CD3 antibody was assessed by tritiated thymidine [^3^H] incorporation. Stimulation index (SI) was calculated by dividing the mean cpm of stimulated T cells (in response to 1 μg/ml anti-CD3) by the mean cpm of unstimulated cells (in media alone). Anti-CD3-stimulated IL-2 **(B)** and IFN-γ **(C)** secretion from T cells was measured by ELISA. Data are presented as mean ± SEM. T-cell proliferation was significantly correlated with IL-2 secretion [**(D)**; Spearman *r* = 0.3751, *p* = 0.0264], but not with IFN-γ secretion [**(E)**; Spearman *r* = 0.0689, *p* = 0.6941].

T-cell proliferation (Figure 1A), IL-2 (Figure 1B), and IFN-γ (Figure 1C) secretion in response to 1 μg/ml anti-CD3 antibody stimulation were measured to determine the effector function of T cells from participants. The mean T-cell proliferation (reported as SI) in response to anti-CD3 antibody was 149.40 ± 19.52 (range 15.04–511.80). The mean IL-2 and IFN-γ production from anti-CD3-stimulated T cells was 0.37 ± 0.08 ng/ml (range 0.00–1.45 ng/ml) and 102.70 ± 8.09 ng/ml (range 1.35–169.40 ng/ml), respectively. We observed a large variation in all T-cell effector functions (proliferation and cytokine secretion). Therefore, the relationship between T-cell proliferation and cytokine secretion was examined to determine if subjects with greater T-cell proliferation were those with high IL-2 and IFN-γ production. T-cell proliferation was significantly correlated with IL-2 secretion (Figure 1D; Spearman *r* = 0.3751, *p* = 0.0264), but not with IFN-γ secretion (Figure 1E; Spearman *r* = 0.0689, *p* = 0.6941).

### Activation marker expression on unstimulated T cells

**Figure 2:**
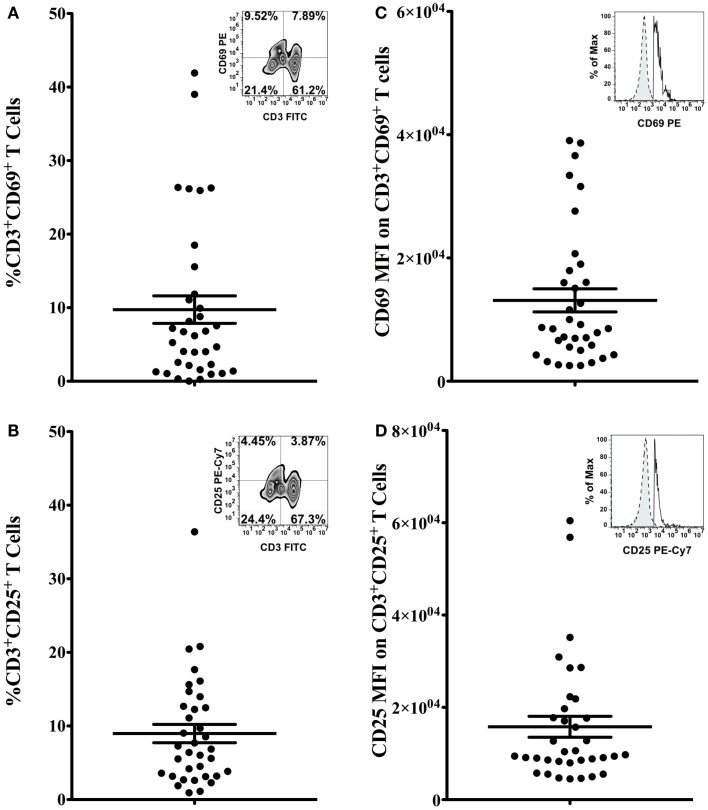
**Activation marker expression on T cells**. Percentage of CD3^+^CD69^+^ T cells **(A)** and CD3^+^CD25^+^ T cells **(B)** in total PBMCs, and the MFI of CD69 on CD3^+^CD69^+^ T cells **(C)** and the MFI of CD25 on CD3^+^CD25^+^ T cells **(D)** was assessed by flow cytometry. Data are presented as mean ± SEM. Bivariate plots of CD3 vs. CD69 expression [**(A)** inset] or CD25 expression [**(B)** inset] from one representative subject are shown. Insets of **(C,D)** show flow histograms of CD69 MFI on CD3^+^CD69^+^ T cells and CD25 MFI on CD3^+^CD25^+^ cells from one representative subject. Dashed lines represent isotype controls and solid lines represent experimental samples.

The percentage of CD3^+^CD69^+^ (Figure 2A) and CD3^+^CD25^+^ (Figure 2B) cells, and the mean fluorescence intensity (MFI) of CD69 and CD25 on double positive cells (Figures 2C,D, respectively) were quantified to determine the activation status of freshly isolated T cells. Bivariate plots of CD3 vs. CD69 expression are shown from one representative subject in Figures 2A,B inset graphs, respectively. The mean percentage of CD3^+^CD69^+^ T cells in PBMCs was 9.74 ± 1.87% (range 0.03–41.92%, Figure 2A), and the mean percentage of CD3^+^CD25^+^ T cells in PBMCs was 8.97 ± 1.24% (range 0.92–36.39%, Figure 2B). The MFI of CD69 on CD3^+^CD69^+^ T cells was 13,119 ± 1878 (range 2546–39,050), and the average MFI of CD25 on CD3^+^CD25^+^ T cells was 15,791 ± 2267 (range 4504–60,450). Representative flow histograms of CD69 expression on CD3^+^CD69^+^ cells and CD25 expression on CD3^+^CD25^+^ cells are shown in Figures 2C,D inset graphs, respectively.

### Activation marker expression on unstimulated T cells was associated with T-cell proliferation and cytokine secretion

To determine if activation marker expression (CD69 and CD25 expression) on freshly isolated, unstimulated T cells and T-cell subsets, and other host factors (e.g., age, BMI, physical activity, and dietary factors) contribute to the variation in effector function (e.g., T-cell proliferation and IL-2 and IFN-γ secretion), linear regression analysis was used. Most of the variables in Table 1 contributed less than 10% to any of the immune outcomes (T-cell proliferation and IL-2 and IFN-γ secretion) when included in the model alone (without activation marker expression), with the exception of age, HDL, and vitamin D which contributed 10.3, 11.0, and 11.3% of the variability in IL-2 secretion (Table 2; *R*^2^ = 10.25; 10.98; and 11.27%, respectively). Also, hs-CRP and vitamin D contributed to 11.6 and 12.1% of the variability in IFN-γ secretion (Table 2; *R*^2^ = 11.55 and 12.13%, respectively). However, the MFI of CD69 on CD3^+^CD69^+^ T cells alone accounted for 29% of the variability in T-cell proliferation (Table 3, model 1, β = 0.0058, *R*^2^ = 29.17%, *p* = 0.0008). When BMI and the MFI of CD69 on unstimulated CD3^+^CD69^+^ T cells were included in the model, 45% of the variability in T-cell proliferation was explained (Table 3, model 2, β = 0.0063, *R*^2^ = 44.95%, *p* < 0.0001). The MFI of CD25 on unstimulated CD3^+^CD25^+^ T cells was also significantly associated with T-cell proliferation. The MFI of CD25 on CD3^+^CD25^+^ T cells alone accounted for 16% of the variability in T-cell proliferation (Table 3, model 1, β = 0.0035, *R*^2^ = 15.78%, *p* = 0.0181). However, the predicted value of CD25 MFI on unstimulated CD3^+^CD25^+^ T cells was no longer significant when physical activity, daily intake of vitamin D, selenium, and n-3 PUFA were included in the model (Table 3, model 2, β = 0.0021, *R*^2^ = 36.29%, *p* = 0.1490). Similar analyses were conducted to explore the relationship between the MFI of CD69 and CD25 on unstimulated T-cell subsets and T-cell proliferation. Data are presented in Tables S1 and S2 in Supplementary Material. The *R*^2^ of the predictor (activation marker expression) plus the individual confounding variables included in the models described in Table 3 were examined to compare the relative contribution of each confounding variable when the predictor was in the model. BMI accounted for 16% of the variability in T-cell proliferation when CD69 MFI on CD3^+^CD69^+^ T cells were included in the model (Table S3 in Supplementary Material; *R*^2^ = 15.79%). Dietary selenium intake accounted for 10% of the variability in T-cell proliferation when CD25 MFI on CD3^+^CD25^+^ T cells was included in the model (Table S3 in Supplementary Material; *R*^2^ = 10.30%).

**Table 2:** **Contributions of individual variables to immune function and cold or flu incidence and severity^a^^,^^b^^,^^c^**.

	*R*^2^ (%)					Odds ratio (95% CI)	95% CI
	SI	IL-2 secretion	IFN-γ secretion	TNF-α secretion	IL-6 secretion	Cold or flu incidence	Number of days with cold or flu
Age	1.38	10.25	2.69	0.01	0.35	1.085 (0.867, 1.359)	(−0.105, 0.120)
BMI	0.72	0.39	2.70	2.81	0.16	1.085 (0.619, 1.901)	(−0.258, 0.240)
Insulin	1.77	3.40	3.24	2.27	0.47	0.789 (0.546, 1.139)	(−0.040, 0.191)
CRP	0.31	3.45	11.55	3.19	1.25	0.908 (0.683, 1.208)	(−0.021, 0.098)
Serum total cholesterol	6.08	0.07	2.80	0.08	0.17	1.042 (0.989, 1.098)	(−0.038, 0.002)
Serum HDL	0.00	10.98	0.15	1.36	1.55	0.950 (0.839, 1.076)	(−0.042, 0.052)
PA	7.28	8.71	0.15	0.13	5.55	1.169 (0.141, 9.669)	(−1.362, 1.275)
Dietary intake of
Total calories	0.00	3.33	0.02	16.68	9.89	0.998 (0.994, 1.002)	(−0.001, 0.001)
Vitamin C	2.97	2.39	1.99	15.48	5.31	1.006 (0.982, 1.031)	(−0.009, 0.014)
Vitamin D	1.98	11.27	12.13	8.05	4.67	1.002 (0.992, 1.012)	(−0.321, 0.095)
Vitamin E	9.84	0.03	3.13	0.31	1.19	1.440 (0.894, 2.320)	(−0.044, 0.013)
Iron	5.53	4.15	4.53	7.63	12.18	1.246 (0.726, 2.141)	(−0.171, 0.103)
Selenium	2.49	5.07	3.23	0.28	2.93	1.047 (0.964, 1.137)	(−0.044, 0.013)
Zinc	3.19	2.78	3.35	2.60	4.77	0.809 (0.364, 1.799)	(−0.361, 0.214)
n-3 PUFA	1.81	0.41	1.44	0.47	5.80	0.013 (0.001, 5.176)	(0.029, 2.920)
Caffeine	0.04	0.28	0.17	0.02	1.26	1.015 (0.992, 1.038)	(−0.024, 0.005)
Alcohol consumption	0.55	0.01	1.16	1.87	2.73	1.039 (0.824, 1.311)	(−0.085, 0.115)

*^a^Waist circumference, glucose, SBP, and DBP were not included because they were collinear with BMI*.

*^b^Serum LDL and TG were not included because they were collinear with serum total cholesterol*.

*^c^Dietary intake of carbohydrates, proteins, and fat were not included because they were collinear with total calorie intake*.

**Table 3:** **Activation marker expression on unstimulated T cells as predictors of T-cell effector function**.

	CD69 MFI on CD3^+^CD69^+^ T cells				CD25 MFI on CD3^+^CD25^+^ T cells			
	β	*R*^2^ (%)	Variables in the model	*p* value	β	*R*^2^ (%)	Variables in the model	*p* value
**Anti-CD3-induced T-cell proliferation^a^**								
Model 1	0.0058	29.17	CD69 MFI on CD3^+^CD69^+^ T cells	0.0008	0.0035	15.78	CD25 MFI on CD3^+^CD25^+^ T cells	0.0181
Model 2	0.0063	44.95	Model 1 + BMI	<0.0001	0.0021	36.29	Model 1 + PA, vitamin D, selenium, n-3 PUFA	0.1490
**Anti-CD3-induced IL-2 secretion from T cells**								
Model 1	0.0242	29.02	CD69 MFI on CD3^+^CD69^+^ T cells	0.0010	0.0276	55.29	CD25 MFI on CD3^+^CD25^+^ T cells	<0.0001
Model 2	0.0205	64.19	Model 1 + age, PA, total calories, vitamin D, iron	0.0002	0.0209	74.00	Model 1 + age, PA, total calories, vitamin D, iron	<0.0001
**Anti-CD3-induced IFN-γ secretion from T cells**								
Model 1	0.9298	5.28	CD69 MFI on CD3^+^CD69^+^ T cells	0.1911	1.2020	12.91	CD25 MFI on CD3^+^CD25^+^ T cells	0.0369
Model 2	0.7203	30.93	Model 1 + age, BMI, PA, total calories, vitamin C, D, selenium, zinc, alcohol	0.4823	0.9655	31.97	Model 1 + vitamin C, D, selenium, n-3 PUFA	0.0958

*^a^T-cell proliferation was evaluated by quantifying tritiated thymidine incorporation following stimulation with anti-CD3 antibodies, and results are reported as a stimulation index. Stimulation index was calculated by dividing the cpm of the anti-CD3-induced T-cell proliferation by unstimulated T cells*.

The MFI of CD69 on unstimulated CD3^+^CD69^+^ T cells was also significantly associated with IL-2 secretion from anti-CD3-stimulated T cells. The MFI of CD69 on CD3^+^CD69^+^ T cells accounted for 29% of the variability in IL-2 secretion (Table 3, model 1, β = 0.0242, *R*^2^ = 29.02%, *p* = 0.0010). When age, physical activity, daily total calories, vitamin D, and iron intake were included in the model as confounding factors, 64% of the variability in IL-2 secretion was explained (Table 3, model 2, β = 0.0205, *R*^2^ = 64.19%, *p* = 0.0002). The MFI of CD25 on unstimulated CD3^+^CD25^+^ T cells was also significantly associated with IL-2 secretion. The MFI of CD25 on freshly isolated, unstimulated CD3^+^CD25^+^ T cells alone accounted for 55% of the variability in IL-2 secretion (Table 3, model 1, β = 0.0276, *R*^2^ = 55.29%, *p* < 0.0001). After controlling for age, daily total calories, vitamin D, and iron intake as confounding factors, the MFI of CD25 on unstimulated CD3^+^CD25^+^ T cells and confounding factors accounted for 74% of the variability in IL-2 secretion (Table 3, model 2, β = 0.0209, *R*^2^ = 74.00%, *p* < 0.0001). Similar analyses were done to explore the relationship between the MFI of CD69 and CD25 on unstimulated T-cell subsets and IL-2 secretion from anti-CD3-stimulated T cells. Data are presented in Tables S1 and S2 in Supplementary Material. The *R*^2^ of the predictor (activation marker expression) plus the individual confounding variables included in the models described in Table 2 were examined to compare the relative contribution of each confounding variable when the predictor was in the model. None of the individual dietary or lifestyle variables contributed more than 10% to variability in IL-2 secretion (Table S3 in Supplementary Material).

Unlike the relationship between T-cell activation marker expression and IL-2 secretion, only the MFI of CD25 on unstimulated CD3^+^CD25^+^ cells was associated with IFN-γ secretion from anti-CD3-stimulated T cells. CD25 MFI on unstimulated CD3^+^CD25^+^ cells alone accounted for 13% of the variability in IFN-γ secretion (Table 3, model 1, β = 1.2020, *R*^2^ = 12.91%, *p* = 0.0369). However, the value of CD25 MFI on unstimulated CD3^+^CD25^+^ cells was no longer significant when daily intake of vitamin C, vitamin D, selenium, and n-3 PUFA were included in the model as confounding variables (Table 3, model 2, β = 0.9655, *R*^2^ = 31.97%, *p* = 0.0958). CD69 MFI on CD3^+^CD69^+^ T cells was not associated with IFN-γ secretion before (Table 3, model 1, β = 0.9298, *R*^2^ = 5.28, *p* = 0.1911) and after adjusting for confounding variables (Table 3, model 2, β = 0.7203, *R*^2^ = 30.93, *p* = 0.4823). Similar analyses were done to explore the relationship between the MFI of CD69 and CD25 on unstimulated T-cell subsets and IFN-γ secretion from anti-CD3-stimulated T cells. Data are presented in Tables S1 and S2 in Supplementary Material. Since the MFI of CD69 and CD25 alone on T cells was not associated with IFN-γ secretion, we further examined the relationship between IFN-γ secretion and other potential immunomodulatory factors, including age, BMI, physical activity level, daily intake of total calories, vitamin C, vitamin D, vitamin E, selenium, iron, zinc, n-3 PUFA, caffeine, and alcohol consumption to determine which variables significantly impacted IFN-γ secretion. Daily intake of vitamin D was the only variable that was significantly associated inversely with IFN-γ secretion. Vitamin D intake contributed to 12% of the variability in IFN-γ secretion (Table 2, *R*^2^ = 12.13; data not shown, β = −88.4027, *p* = 0.0470).

### Cytokine secretion and HLA-DR expression on DCs and macrophages

The percentage of DCs (CD123^+^CD11c^+^HLA-DR^+^) and macrophages (CD14^+^HLA-DR^+^) in PBMCs, and HLA-DR expression on unstimulated DCs and macrophages were both quantified. The percentage of DCs in PBMCs was 4.86 ± 1.12% (range 0.07–14.18%) and the average MFI of HLA-DR on DCs was 94,446 ± 10,084 (range 23,500–255,000; data not shown). The percentage of macrophages in PBMCs was 14.38 ± 1.00% (range 3.79–27.22%) and the average MFI of HLA-DR on macrophages was 46,817 ± 3528 (range 16,100–111,000; data not shown). TNF-α and IL-6 secretion from PBMCs in response to 10 μg/ml LPS stimulation was also measured. The mean TNF-α and IL-6 production from LPS-stimulated PBMCs was 1.16 ± 0.10 ng/ml (range 0.33–2.39 ng/ml) and 15.01 ± 1.15 ng/ml (range 5.55–25.05 ng/ml), respectively. Of the variables shown in Table 1, dietary iron contributed to 12.2% of the variability in IL-6 (Table 2; *R*^2^ = 12.18%) and total calories and vitamin C contributed to 16.7 and 15.5% of the variability in TNF-α secretion (Table 2; *R*^2^ = 16.68 and 15.48%, respectively). All other variables contributed to less than 10% of the variability in IL-6 and TNF-α secretion. HLA-DR expression on DCs and macrophages was not associated with TNF-α and IL-6 production from LPS-stimulated DCs and macrophages (data not shown).

### Cytokine secretion was associated with the incidence and severity of cold or flu symptoms

**Figure 3:**
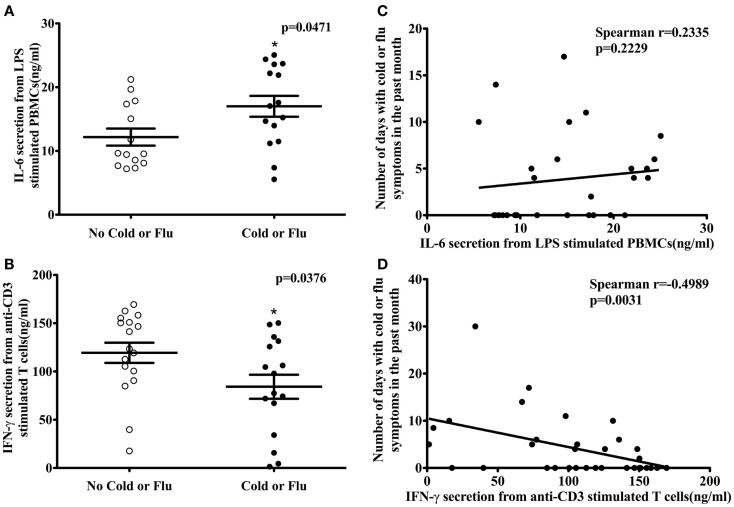
**LPS-stimulated IL-6 secretion from PBMCs, anti-CD3-stimulated IFN-γ secretion from T cells, and self-reported cold or flu status in human subjects**. IL-6 secretion from LPS-stimulated PBMCs was significantly higher in subjects with self-reported cold or flu symptoms compared to subjects without cold or flu symptoms [**(A)**; Mann–Whitney test, *p* = 0.0471]. IL-6 secretion was not correlated with number of days with cold or flu symptoms [**(C)**; Spearman *r* = 0.2335, *p* = 0.2229]. IFN-γ secretion from anti-CD3-stimulated T cells in subjects with cold or flu symptoms was significantly lower compared to subjects without cold or flu symptoms [**(B)**; unpaired *t*-test, *p* = 0.0376]. T-cell IFN-γ secretion was inversely associated with total number of days with self-reported cold or flu symptoms [**(D)**; Spearman *r* = −0.4989, *p* = 0.0031]. Asterisk indicates a significant difference from no cold or flu group (*p* < 0.05).

Participants (*n* = 34) completed a URTI questionnaire at their clinical visit. According to their self-reported cold or flu incidence, 17 participants had one or more cold or flu episodes in the past month. The association between activation marker expression (CD69 and CD25); T-cell proliferation; T-cell cytokine secretion (IL-2 and IFN-γ); HLA-DR expression on antigen-presenting cells; cytokine secretion (TNF-α and IL-6) from LPS-stimulated PBMCs, and self-reported cold and flu incidence or severity was examined by logistic and Poisson regression, respectively. No association was observed between activation marker expression, T-cell proliferation, HLA-DR expression on antigen-presenting cells, and TNF-α secretion from LPS-stimulated PBMCs and cold and flu incidence and severity (data not shown). However, IL-6 secretion from LPS-stimulated PBMCs was significantly higher in participants with self-reported cold or flu symptoms compared to participants without cold or flu symptoms in the past month (Figure 3A, *p* = 0.0471). The average concentration of IL-6 secretion from LPS-stimulated PBMCs of participants without self-reported cold or flu episodes was 12.17 ± 1.34 ng/ml and the concentration of IL-6 secretion from participants with cold or flu episodes was 17.00 ± 1.64 ng/ml. IL-6 secretion was associated with the incidence of cold or flu episodes before [Table 4, model 1, β = −0.1471, odds ratio (OR) = 0.863, 95% CI = (0.749, 0.994), *p* = 0.0414] and after incorporating daily intake of total calories, vitamin C, iron, and zinc as confounding variables in the model [Table 4, model 2, β = −0.2392, OR = 0.787, 95% CI = (0.632, 0.980), *p* = 0.0325]. However, IL-6 secretion was not associated with the total number of days with self-reported cold or flu symptoms before [Figure 3C, Spearman *r* = 0.2335, *p* = 0.2229; and Table 4, model 1, β = 0.0252, 95% CI = (−0.042, 0.092), *p* = 0.4609] and after incorporating daily intake of total calories, vitamin D, iron, and zinc as confounding variables in the model [Table 4, model 2, β = 0.0801, 95% CI = (−0.018, 0.178), *p* = 0.1105].

**Table 4:** **Cytokine secretion as predictors of self-reported cold or flu incidence and severity**.

	LPS-induced IL-6 secretion from PBMCs				Anti-CD3-induced IFN-γ secretion from T cells			
	β	Odds ratio (95% CI)	Variables in the model	*p* value	β	Odds ratio (95% CI)	Variables in the model	*p* value
**Incidence of cold or flu episode in the past month^a^^,^^b^**								
Model 1	−0.1471	0.863 (0.749, 0.994)	IL-6 secretion	0.0414	0.0141	1.014 (0.997, 1.032)	IFN-γ secretion	0.1019
Model 2	−0.2392	0.787 (0.632, 0.980)	Model 1 + total calories, vitamin C, iron, zinc	0.0325	0.0556	1.057 (0.986, 1.133)	Model 1 + age, BMI, vitamin D, iron, zinc, n-3 PUFA, alcohol, caffeine	0.1158
		
	**β**	**95% CI**	**Variables in the model**	***p* value**	**β**	**95% CI**	**Variables in the model**	***p* value**

**Total number of days with cold or flu symptoms in the past month^c^**								
Model 1	0.0252	−0.042, 0.092	IL-6 secretion	0.4609	−0.0138	−0.022, −0.006	IFN-γ secretion	0.0005
Model 2	0.0801	−0.018, 0.178	Model 1 + total calories, iron, zinc	0.1105	−0.0153	−0.021, −0.009	Model 1 + vitamin C, zinc, alcohol, caffeine	<0.0001

*^a^Logistic regression model is fitted to determine the relationship between IL-6 or IFN-γ secretion and incidence of cold or flu in the past month*.

*^b^No cold or flu episode in the past month = 0, presence of a cold or flu episode in the past month = 1. Probability modeled is no cold or flu episode in the past month*.

*^c^Poisson regression model is fitted to determine the relationship between IL-6 or IFN-γ secretion and total number of days with cold or flu symptoms in the past month*.

In contrast to IL-6 secretion, IFN-γ secretion from anti-CD3-stimulated T cells was significantly lower in participants with self-reported cold or flu symptoms compared to participants without these symptoms in the past month (Figure 3B, *p* = 0.0376). The average concentration of IFN-γ secretion from anti-CD3-stimulated T cells of participants without self-reported cold or flu episodes was 119.30 ± 10.45 ng/ml and the concentration of IFN-γ secretion from participants with cold or flu episodes was 84.19 ± 12.45 ng/ml. IFN-γ secretion was not associated with the self-reported incidence of cold or flu episodes alone [Table 4, model 1, β = 0.0141, OR = 1.014, 95% CI = (0.997, 1.032), *p* = 0.1019], or after incorporating confounding variables (age, BMI, daily intake of vitamin D, iron, zinc, n-3 PUFA, caffeine, and alcohol consumption) in the model [Table 4, model 2, β = 0.0556, OR = 1.057, 95% CI = (0.983, 1.133), *p* = 0.1158]. However, IFN-γ secretion was inversely correlated with total number of days with self-reported cold or flu symptoms (Figure 3D, Spearman *r* = −0.4989, *p* = 0.0031). IFN-γ secretion was significantly associated with total number of days with cold or flu symptoms [Table 4, model 1, β = −0.0138, 95% CI = (−0.022, −0.006), *p* = 0.0005]. After incorporating daily intake of vitamin C, zinc, caffeine, and alcohol consumption as confounding variables in the model, IFN-γ secretion was also significantly associated with total number of days with cold or flu symptoms [Table 4, model 2, β = −0.0153, 95% CI = (−0.021, −0.009), *p* < 0.0001]. The contributions of the predictor (cytokine secretion) and individual confounding variables in the regression models with cold or flu incidence and severity as dependent variables are included as Table S4 in Supplementary Material. Individual variables contributed a small amount to the model (e.g., prediction of cold or flu incidence or total number of days with cold symptoms). However, cytokine secretion better predicted the incidence or severity of cold of flu when dietary factors were included in the models.

## Discussion

To our knowledge, this study provides the first documentation that expression of activation markers on freshly isolated, unstimulated T cells is associated with effector function of activated T cells from healthy human subjects. Flow cytometric analysis of CD69 and CD25 expression on unstimulated T cells and T-cell subsets was significantly correlated with anti-CD3-stimulated T-cell proliferative response and IL-2 secretion. However, neither CD69 nor CD25 expression was associated with IFN-γ secretion. These data suggest that CD69 and CD25 expression on unstimulated T cells may be a useful predictor of T-cell effector function following stimulation. Furthermore, host factors (e.g., age, BMI, physical activity, total calories, and select dietary factors) contribute significantly to the relationship between activation marker expression and T-cell effector function. Our study is also the first to demonstrate that LPS-stimulated IL-6 secretion from PBMCs and anti-CD3-induced IFN-γ secretion from T cells were significantly associated with self-reported incidence and severity of cold or flu in the past month, respectively. These findings demonstrate the incidence and severity of cold or flu symptoms captured via the URTI questionnaire were accompanied by relevant immunological changes. This relationship was also strengthened by the inclusion of host factors in the model. Collectively, these data suggest lifestyle and dietary factors are important variables that contribute to the immune response and should be included in human clinical trials assessing immune endpoints.

Few clinical studies have examined T-cell proliferation and IL-2 and IFN-γ secretion in response to anti-CD3 stimulation in young healthy adults. Results from our study demonstrate that among this population, T-cell effector function varied greatly, with some individuals demonstrating robust T-cell effector responses, and others not. To determine if these individuals with high T-cell proliferation also had high cytokine secretion, we examined the correlation between T-cell proliferation and IL-2 and IFN-γ secretion. T-cell proliferation was significantly correlated with IL-2 secretion but not IFN-γ secretion. These results are not surprising given the essentiality of IL-2 for T-cell proliferation (32). Previous reports have demonstrated a positive association between IFN-γ secretion and T-cell proliferation (33), however, in our population, this was not the case. This may be due to the fact that PBMCs rather than purified T cells were used in the IFN-γ secretion assay. Although T cells are the major sources of IFN-γ secretion, the secretion of IFN-γ by other immune cells (e.g., B cells, nature killer cells) may minimize the association between IFN-γ secretion and T-cell proliferation (34). The limited sample size (*n* = 36) in our study may also partly explain the inconsistency between our results and previous studies.

In addition to the heterogeneity in T-cell effector responses, we observed that activation marker expression on T cells also varied greatly among subjects. Stimulation of T cells by antigens or mitogens induces upregulation of CD69 within 4 h, and CD25 between 24 and 48 h following T-cell activation, which in turn triggers downstream signaling pathways that initiates proliferation and differentiation of T cells (32, 35–38). Several previous studies have reported that CD69 or CD25 expression on antigen- or mitogen-stimulated T cells is up-regulated in parallel with T-cell proliferation (determined by [^3^H] thymidine incorporation) (39–41). However, these results are not consistent across all studies (39–41). Results vary based on the different types of stimuli (antigen or mitogen) and the length of the assay (39–41). Simms et al. reported that anti-CD3 or staphylococcal enterotoxin (SEB)-induced T-cell proliferation 72 h post stimulation is correlated with the percentage of CD69^+^ T cells 24 h following stimulation (39). Similar results are observed by Maino and colleagues, who found that the percentage of CD3^+^CD69^+^T cells 4 h post activation was correlated with PBMC proliferation following stimulation with anti-CD2 receptor antibodies for 72 h (40). However, there was no correlation between the percentage of CD69^+^ or CD25^+^ T cells and T-cell proliferation induced by various other stimuli [phytohemagglutinin (PHA), SEB, tetanus toxoid, or influenza A virus] for longer time periods (72, 120, 168, and 168 h, respectively) (41). Thus, the nature of mitogenic or antigenic stimuli and the time course of activation influence the relationship between activation marker expression on T cells and proliferative response of T cells. These studies suggest that quantification of CD69 and CD25 expression on stimulated T cells may not be a consistent marker of T-cell proliferative capacity because the variability in assay conditions confounds this relationship.

To determine if the activation marker expression on freshly isolated, unstimulated T cells was predictive of T-cell proliferation, we used linear regression analyses using the expression of CD69 or CD25 (MFI of CD69 on CD3^+^CD69^+^ or MFI of CD25 on CD3^+^CD25^+^) on unstimulated T cells as the independent variable (predictor) and anti-CD3-induced T-cell proliferation as the dependent variable (outcome). The MFI of CD69 on unstimulated CD3^+^CD69^+^ T-cell contributed significantly (29.2%) to the variability in T-cell proliferation (Table 3). To further explain the heterogeneity in T-cell proliferation, we included other host variables in the model that have previously been reported to modulate T-cell proliferation such as age, BMI, total calories, and select dietary factors (5, 19, 20). The final model included both the MFI of CD69 on CD3^+^CD69^+^ T cells and BMI, and these two variables explained 45% of the variability in T-cell proliferation. Our data demonstrate that CD69 MFI on unstimulated CD3^+^CD69^+^ T cells was predictive of T-cell proliferation in response to anti-CD3 stimulation in young healthy adults. Numerous studies report a co-stimulatory role of CD69 in T-cell proliferation (32, 35–38, 42). Crosslinking CD69 with antibodies enhances human T-cell proliferation induced by phorbol myristate acetate (PMA), PHA, or anti-CD3 antibody (35–38, 42). Thus, baseline differences in CD69 expression may contribute to differences in T-cell proliferation via varying amounts of co-stimulation. Moreover, we found that the BMI of subjects contributed to the heterogeneity of T-cell proliferation, and confounds the association between CD69 expression and T-cell proliferation. Therefore, BMI should be quantified and controlled for in studies quantifying T-cell proliferation.

The MFI of CD25 on unstimulated CD3^+^CD25^+^ T-cell also contributed significantly (16%) to the variability in T-cell proliferation (Table 3). These results are consistent with previous studies which demonstrate a co-stimulatory role of CD25 in T-cell proliferation (32). However, in our study, the MFI of CD25 on CD3^+^CD25^+^ T cells was no longer associated with T-cell proliferation after incorporating physical activity and daily intake of selenium, vitamin D, and n-3 PUFA as confounding variables in our model (3, 18, 21–25). These data suggest that CD25 MFI may only be marginally predictive of T-cell proliferation, but other lifestyle and dietary patterns may contribute significantly to T-cell proliferative responses. Similar analyses using activation marker expression on unstimulated CD4^+^ and CD8^+^ T-cell subsets as predictors find consistent results, which confirm our current observations (Tables S1 and S2 in Supplementary Material).

Similar linear regression analyses were performed to explore the association between CD69 and CD25 expression on unstimulated T cells and anti-CD3-induced IL-2 secretion from T cells. The MFI of CD69 on unstimulated CD3^+^CD69^+^ T-cell contributed significantly (29%) to the variability in IL-2 secretion (Table 3). To further explain the heterogeneity in IL-2 secretion from activated T cells, we included several confounding variables in the model that have previously been reported to modulate IL-2 secretion (3, 16, 18, 19, 21, 22, 26–31). The final model included both the MFI of CD69 on CD3^+^CD69^+^ T cells and age, physical activity, and daily intake of total calories, vitamin D, and iron. Combined, these variables explained 64% of the variability in IL-2 secretion. Our observations are consistent with previous studies, which report that crosslinking CD69 with anti-CD69 antibodies enhance human IL-2 secretion following T-cell activation induced by PMA, PHA, or anti-CD3 antibody (35–38, 42). However, our findings are novel because we demonstrate that individual lifestyle and dietary variables contribute a small amount to IL-2 secretion. However, these variables significantly improved the predictive relationship between CD69 expression and IL-2 secretion suggesting that host factors are important variables to quantify in clinical studies assessing T-cell function.

Similarly, the MFI of CD25 on unstimulated CD3^+^CD25^+^ T-cell contributed significantly (55%) to the variability in IL-2 secretion (Table 3). In our final model, we controlled for age, physical activity, and daily intake of total calories, vitamin D, and iron, and the MFI of CD25 on unstimulated CD3^+^CD25^+^ T cells and together these variables contributed significantly (74%) to the variability in IL-2 secretion. Our findings are not surprising given the autocrine loop formed between CD25 expression and IL-2 secretion during T-cell activation (32). Similar analyses using activation marker expression on unstimulated CD4^+^ and CD8^+^ T-cell subsets as predictors found consistent results, which confirmed our current observations (Tables S1 and S2 in Supplementary Material). Our data suggest that the MFI of CD25 on unstimulated CD3^+^CD25^+^ T cells could serve as predictor of IL-2 secretion from T cells in response to anti-CD3 stimulation in young healthy adults. In addition, we found that host factors including age, physical activity, daily intake of total calories, vitamin D, and iron of subjects contributed to the association between the MFI of CD25 on unstimulated CD3^+^CD25^+^ T cells and IL-2 secretion.

Unlike IL-2 secretion, we did not observe an association between anti-CD3-induced IFN-γ secretion from T cells and the MFI of CD69 on unstimulated CD3^+^CD69^+^ T cells either alone, or after controlling for host factors that have previously been reported to modulate T-cell IFN-γ secretion (22–25, 30, 31). However, the MFI of CD25 on unstimulated CD3^+^CD25^+^ T cells contributed significantly (13%) to the variability in IFN-γ secretion (Table 3). Previous studies demonstrate that the interaction of IL-2 with CD25 (α chain of the IL-2 receptor) induces T cells to secrete IFN-γ (32, 33). These data support the association between MFI of CD25 alone and IFN-γ secretion. However, in our study the association between CD25 MFI on unstimulated CD3^+^CD25^+^ T cells and IFN-γ secretion was no longer significant after controlling for daily intake of selenium, vitamin C, vitamin D, and n-3 PUFA suggesting that these dietary factors significantly influence IFN-γ response in human T cells (22–25, 30, 31). In fact, of the aforementioned dietary variables, vitamin D intake contributed to 12% of the variability in IFN-γ secretion, and daily intake of vitamin D was inversely associated with IFN-γ secretion. This finding is in agreement with previous studies, which report that vitamin D (25-hydroxyvitamin D) and 1,25 dihydroxyvitamin D3, the active form of vitamin D, inhibit IFN-γ secretion from mitogen-stimulated T cells in humans and mice (24). Results from our study suggest that activation marker expression (either CD69 or CD25) on freshly isolated, unstimulated T cells was not strongly predictive of IFN-γ secretion from activated T cells. Moreover, dietary and lifestyle factors (in particular vitamin D status) contributed significantly to the variability in IFN-γ secretion. Thus, the relationship between dietary and lifestyle factors and IFN-γ secretion needs to be examined in a larger study to confirm these findings and to determine the magnitude of the relationship. Similar analyses using activation marker expression on unstimulated CD4^+^ and CD8^+^ T-cell subsets as predictors found consistent results (Tables S1 and S2 in Supplementary Material).

To determine if the incidence and severity of cold or flu symptoms captured via the self-reported URTI questionnaire is accompanied by relevant immunological changes, we used two analytical strategies. First, we performed logistic regression analysis using IL-6 secretion from LPS-stimulated PBMCs as the independent variable (predictor) and incidence of cold or flu episodes as the dependent variable (outcome). Next, we ran Poisson regression analysis using IL-6 secretion from LPS-stimulated PBMCs as the independent variable (predictor) and total number of days with cold or flu symptoms as the dependent variable (outcome). From these two approaches, we found that IL-6 secretion was higher in participants with self-reported cold or flu episodes compared to participants without cold or flu episodes, and was significantly associated with cold or flu incidence in the past month (Figure 3; Table 4). IL-6 secretion remained significantly associated with incidence of cold or flu after incorporating total calories, vitamin C, iron, and zinc in the model (Table 4). However, we did not observe an association between total number of days with cold or flu symptoms and IL-6 secretion alone and with the addition of confounding variables (daily intake of total calories, iron, and zinc). Our findings suggest that IL-6 secretion from LPS-stimulated PBMCs was associated with the incidence of cold or flu episodes, but was not related to the severity of cold or flu symptoms captured via self-reported URTI questionnaire. In addition, we found that when dietary factors (total calories, vitamin C, iron, and zinc) were included in the model, the association between IL-6 secretion and the incidence of cold or flu symptoms in young healthy adults was strengthened.

Previous studies report elevated levels of IL-6 during cold or flu infection as a result of ongoing inflammation (43–45). The release of IL-6 during cold or flu is also critical in coordinating the innate and adaptive immune responses for efficient clearance of infection (45). The heterogeneity in IL-6 secretion from PBMCs in response to LPS stimulation may be attributable to genetic and environmental factors (46, 47). Previous studies demonstrate an association between polymorphisms in the gene encoding IL-6 (G/C polymorphism was detected at position-174) and secreted level of IL-6 following stimulation. In particular, homozygosity of IL-6-174C allele is associated with reduced transcription and secretion of IL-6 in human plasma (48) and with decreased frequency of the common cold (46). Therefore, the heterogeneity in LPS-stimulated IL-6 secretion from PBMCs observed in our study may be due to polymorphisms in the gene encoding IL-6, as well as environmental factors (e.g., BMI, dietary intake, and physical activity), which contribute to the difference in IL-6 secretion between participants with and without self-reported cold or flu.

Similar logistic and Poisson regression analyses were also performed to determine if the incidence and severity of cold or flu symptoms captured via the self-reported URTI questionnaire is accompanied by IFN-γ secretion from anti-CD3-stimulated T cells. The incidence of cold or flu was not associated with IFN-γ secretion alone or following inclusion of age, BMI, daily intake of vitamin D, iron, zinc, n-3 PUFA, caffeine, and alcohol consumption in the model (Table 4). However, IFN-γ secretion from anti-CD3-stimulated T cells was significantly associated with total number of days with cold or flu symptoms alone and with the addition of daily intake of vitamin C, zinc, caffeine, and alcohol consumption (Table 4). Our results suggest that the severity but not the incidence of cold or flu symptoms assessed via the self-reported URTI questionnaire was accompanied by changes in IFN-γ secretion from anti-CD3-stimulated T cells in healthy young adults. We also observed that the association between IFN-γ secretion and severity of cold or flu symptoms was confounded by daily intake of vitamin C, zinc, caffeine, and alcohol consumption, suggesting that these dietary components impact this relationship.

Interferon-gamma production has been reported to play an important role in the immune response against numerous viral infections (47, 49). Thus, individuals who have lower IFN-γ production may be more susceptible to infection with influenza virus or rhinoviruses causing the common cold. The heterogeneity in IFN-γ secretion following T-cell activation that we observed may also be attributable to genetic and environmental factors (46, 47). For example, a single nucleotide polymorphism (SNP) in IFN-γ gene has been studied extensively and is presented as polymorphism +874 A/T. Results from a meta-analysis demonstrate that the variant allele +874 A is correlated with lower levels of IFN-γ production and increased risk of *Mycobacterium tuberculosis* infection (50). It is plausible that individuals carrying the +874A allele may be susceptible to other infections, including influenza infection. Thus, IFN-γ gene polymorphisms may contribute to the differences in IFN-γ secretion between individuals with and without cold or flu episodes and the association between IFN-γ secretion and incidence and severity of cold or flu symptoms in our study. However, Becker et al. reported no association between common cold frequency and CD2-induced IFN-γ secretion from T cells in adults 45–65 years old (49). Previous studies demonstrate an age-related reduction in the synthesis and secretion of IFN-γ from PBMCs (26, 27). Thus, age may also impact the relationship between cold and flu incidence and IFN-γ secretion.

In summary, we demonstrated that expression of CD69 and CD25 expression on freshly isolated, unstimulated T cells was significantly associated with anti-CD3-stimulated T-cell proliferation and IL-2 secretion. We also found that IL-6 secretion from LPS-stimulated PBMCs was associated with self-reported incidence of cold or flu episodes, and IFN-γ secretion from T cells was associated with self-reported severity of cold or flu symptoms in the past month. Our data suggest that the incidence and severity of cold or flu symptoms captured via the URTI questionnaire was accompanied by relevant immunological changes. In addition, we demonstrated that host-related factors, including age, BMI, physical activity, total calorie intake, and various dietary components contributed to heterogeneity in T-cell function and incidence and severity of cold or flu infection. These factors also confounded the association between activation marker expression on T cells and T-cell effector function, and the association between innate and adaptive immune response and incidence and severity of cold or flu symptoms. Therefore, quantification of dietary factors in human clinical trials measuring immune function may be crucial to understanding the variability in immune responses among subjects, and to determine the true relationship between an intervention of interest and immune outcomes.

## Conflict of Interest Statement

The authors declare that the research was conducted in the absence of any commercial or financial relationships that could be construed as a potential conflict of interest.

## Supplementary Material

The Supplementary Material for this article can be found online at http://www.frontiersin.org/Journal/10.3389/fimmu.2015.00094/abstract

## Abbreviations

BMI, body mass index; Con A, concanavalin A; DCs, dendritic cells; HDL, high-density lipoprotein; hs-CRP, high-sensitivity C-reactive protein; IFN-γ, interferon-gamma; IL-2, interleukin-2; IL-6, interleukin-6; IPAQ, International Physical Activity Questionnaires; LDL, low-density lipoprotein; LPS, lipopolysaccharide; MET, metabolic equivalent of task; MFI, mean fluorescence intensity; n-3 PUFA, n-3 polyunsaturated fatty acids; OR, odds ratio; PBMC, peripheral blood mononuclear cells; PHA, phytohemagglutinin; PMA, phorbol myristate acetate; SEB, staphylococcal enterotoxin; SI, stimulation index; TC, total cholesterol; TG, triglycerides; TNF-α, tumor necrosis factor-alpha; TT, tetanus toxoid; URTI, upper respiratory tract infection.
