# Supplementary material for: In vitro Production of IL-6 and IFN-γ is Influenced by Dietary Variables and Predicts Upper Respiratory Tract Infection Incidence and Severity Respectively in Young Adults
Source: Front Immunol. 2015 Mar 4;6:94. doi: 10.3389/fimmu.2015.00094 (PMC4349184; doi:10.3389/fimmu.2015.00094)
Supplement: Supplementary file 1 [file Table_1.DOCX]

**Supplement table 1. CD69 and CD25 expression on unstimulated CD4^+^ T cell as predictors of T cell effector function.**

|  | **CD69 MFI on CD3^+^CD4^+^CD69^+^ T cells** | | | | |  | **CD25 MFI on CD3^+^CD4^+^CD25^+^ T cells** | | | |
| --- | --- | --- | --- | --- | --- | --- | --- | --- | --- | --- |
|  | **β** | | **R^2^ (%)** | **Variables in the model** | **p value** |  | **β** | **R^2^ (%)** | **Variables in the model** | **p value** |
| **Anti-CD3 induced T cell proliferation^1^** | | | | | |  |  | | | |
| Model 1 | 0.00584 | 27.02 | | CD69 MFI on CD3^+^CD4^+^CD69^+^ T cells | 0.0014 |  | 0.00899 | 16.67 | CD25 MFI on CD3^+^CD4^+^CD25^+^ T cells | 0.0149 |
| Model 2 | 0.00681 | 45.44 | | Model 1 + BMI | <0.0001 |  | 0.00587 | 35.89 | Model 1 + PA, selenium, n-3 PUFA | 0.1065 |
| **Anti-CD3 induced IL-2 secretion from T cells** | | | | |  |  |  | | | |
| Model 1 | 0.02571 | 29.51 | | CD69 MFI on CD3^+^CD4^+^CD69^+^ T cells | 0.0009 |  | 0.05469 | 34.94 | CD25 MFI on CD3^+^CD4^+^CD25^+^ T cells | 0.0002 |
| Model 2 | 0.02011 | 54.36 | | Model 1 + age, total calories, vitamin D, zinc, iron | 0.0051 |  | 0.04065 | 69.68 | Model 1 + age, PA, total calories, vitamin D, zinc, iron | 0.0013 |
| **Anti-CD3 induced IFN-ɣ secretion from T cells** | | | | |  |  |  | | | |
| Model 1 | 1.43915 | 11.36 | | CD69 MFI on CD3^+^CD4^+^CD69^+^ T cells | 0.0512 |  | 2.68772 | 10.36 | CD25 MFI on CD3^+^CD4^+^CD25^+^ T cells | 0.0634 |
| Model 2 | 1.22687 | 30.84 | | Model 1 + BMI, vitamin C, D, selenium, n-3 PUFA | 0.1385 |  | 2.37393 | 38.13 | Model 1 + vitamin D, E, selenium, zinc, iron, n-3 PUFA | 0.1273 |

^1^ T cell proliferation was evaluated by quantifying tritiated thymidine incorporation following stimulation with anti-CD3 antibodies, and results are reported as a stimulation index. Stimulation index was calculated by dividing the cpm of the anti-CD3 induced T cell proliferation by unstimulated T cells.
