# Supplementary material for: In vitro Production of IL-6 and IFN-γ is Influenced by Dietary Variables and Predicts Upper Respiratory Tract Infection Incidence and Severity Respectively in Young Adults
Source: Front Immunol. 2015 Mar 4;6:94. doi: 10.3389/fimmu.2015.00094 (PMC4349184; doi:10.3389/fimmu.2015.00094)
Supplement: Supplementary file 2 [file Table_2.DOCX]

**Supplement table 2. CD69 and CD25 expression on unstimulated CD8^+^ T cell as predictors of T cell effector function.**

|  | | **CD69 MFI on CD3^+^CD8^+^CD69^+^ T cells** | | | | |  | **CD25 MFI on CD3^+^CD8^+^CD25^+^ T cells** | | | |
| --- | --- | --- | --- | --- | --- | --- | --- | --- | --- | --- | --- |
|  |  | **β** | | **R^2^ (%)** | **Variables in the model** | **p value** |  | **β** | **R^2^ (%)** | **Variables in the model** | **p value** |
| **Anti-CD3 induced T cell proliferation^1^** | | | | | | |  |  | | | |
| Model 1 | 0.00604 | | 38.23 | | CD69 MFI on CD3^+^CD8^+^CD69^+^ T cells | <0.0001 |  | 0.00145 | 13.72 | CD25 MFI on CD3^+^CD8^+^CD25^+^ T cells | 0.0285 |
| Model 2 | 0.00764 | | 61.85 | | Model 1 + BMI, vitamin C | <0.0001 |  | 0.00086 | 33.57 | Model 1 + PA, selenium, n-3 PUFA | 0.2023 |
| **Anti-CD3 induced IL-2 secretion from T cells** | | | | | |  |  |  | | | |
| Model 1 | 0.02307 | | 31.56 | | CD69 MFI on CD3^+^CD8^+^CD69^+^ T cells | 0.0005 |  | 0.01196 | 52.64 | CD25 MFI on CD3^+^CD8^+^CD25^+^ T cells | <0.0001 |
| Model 2 | 0.02235 | | 37.72 | | Model 1+age | 0.0006 |  | 0.00866 | 66.63 | Model 1 + age, PA, total calories, vitamin D, iron | 0.0008 |
| **Anti-CD3 induced IFN-ɣ secretion from T cells** | | | | | |  |  |  | | | |
| Model 1 | 0.67363 | | 03.31 | | CD69 MFI on CD3^+^CD8^+^CD69^+^ T cells | 0.3034 |  | 0.51000 | 11.76 | CD25 MFI on CD3^+^CD8^+^CD25^+^ T cells | 0.0471 |
| Model 2 | 0.10972 | | 41.83 | | Model 1 + age, BMI, PA, total calories, vitamin C, E, selenium, zinc, iron, n-3 PUFA, caffeine, alcohol | 0.9145 |  | 0.51000 | 11.76 | Model 1 | 0.0471 |

^1^ T cell proliferation was evaluated by quantifying tritiated thymidine incorporation following stimulation with anti-CD3 antibodies, and results are reported as a stimulation index. Stimulation index was calculated by dividing the cpm of the anti-CD3 induced T cell proliferation by unstimulated T cells.
