# Supplementary material for: In vitro Production of IL-6 and IFN-γ is Influenced by Dietary Variables and Predicts Upper Respiratory Tract Infection Incidence and Severity Respectively in Young Adults
Source: Front Immunol. 2015 Mar 4;6:94. doi: 10.3389/fimmu.2015.00094 (PMC4349184; doi:10.3389/fimmu.2015.00094)
Supplement: Supplementary file 3 [file Table_3.DOCX]

**Supplemental Table 3. Individual R^2^ (%) of predictor (activation marker expression) and confounding variables in the linear regression models with T cell proliferation, IL-2 secretion and IFN-γ secretion as outcome variables.**

|  | T cell proliferation | |  | IL-2 secretion | | | IFN-γ secretion | |
| --- | --- | --- | --- | --- | --- | --- | --- | --- |
|  | CD69 MFI on CD3^+^CD69^+^ T cells | CD25 MFI on CD3^+^CD25^+^ T cells |  | CD69 MFI on CD3^+^CD69^+^ T cells | CD25 MFI on CD3^+^CD25^+^ T cells |  | CD69 MFI on CD3^+^CD69^+^ T cells | CD25 MFI on CD3^+^CD25^+^ T cells |
| Total R^2^ | 44.96 | 36.29 |  | 64.19 | 74.00 |  | 30.94 | 31.97 |
| Predictor | 29.17 | 15.53 |  | 34.72 | 55.73 |  | 1.71 | 12.91 |
| Age |  |  |  | 8.40 | 4.47 |  | 3.68 |  |
| BMI | 15.79 |  |  |  |  |  | 2.49 |  |
| PA |  | 4.41 |  | 3.26 | 5.30 |  | 0.84 |  |
| Total Calories |  |  |  | 7.09 | 2.62 |  | 1.98 |  |
| Vitamin C |  |  |  |  |  |  | 0.73 | 2.33 |
| Vitamin D |  | 0.70 |  | 5.84 | 3.48 |  | 12.13 | 10.02 |
| Vitamin E |  |  |  |  |  |  |  |  |
| Selenium |  | 10.30 |  |  |  |  | 1.53 | 2.92 |
| Zinc |  |  |  |  |  |  | 1.07 |  |
| Iron |  |  |  | 4.88 | 2.40 |  |  |  |
| n-3 PUFA |  | 5.35 |  |  |  |  |  | 3.79 |
| Alcohol |  |  |  |  |  |  | 4.78 |  |
| Caffeine |  |  |  |  |  |  |  |  |
