# Supplementary material for: In vitro Production of IL-6 and IFN-γ is Influenced by Dietary Variables and Predicts Upper Respiratory Tract Infection Incidence and Severity Respectively in Young Adults
Source: Front Immunol. 2015 Mar 4;6:94. doi: 10.3389/fimmu.2015.00094 (PMC4349184; doi:10.3389/fimmu.2015.00094)
Supplement: Supplementary file 4 [file Table_4.DOCX]

**Supplemental Table 4. Individual contribution of predictor (cytokine secretion) and confounding variables in the regression models with cold/flu incidence and severity as outcome variables.**

| Dependent variable | Cold or flu incidence (Odds ratio (95% CI)) | |  | Cold or flu severity (95% CI) | |
| --- | --- | --- | --- | --- | --- |
|  | IL-6 secretion | IFN-γ secretion |  | IL-6 secretion | IFN-γ secretion |
| Predictor | 0.787 (0.632, 0.980) | 1.057 (0.986, 1.133) |  | (-0.018,0.178) | (-0.021, -0.009) |
| Age |  | 1.232 (0.946, 1.604) |  |  |  |
| BMI |  | 0.708 (0.364, 1.378) |  |  |  |
| PA |  |  |  |  |  |
| Total calories | 1.001 (0.999, 1.003) |  |  | (-0.001, 0.000) |  |
| Vitamin C | 0.997 (0.979, 1.015) |  |  |  | (0.004, 0.016) |
| Vitamin D |  | 1.008 (0.997, 1.020) |  |  |  |
| Vitamin E |  |  |  |  |  |
| Selenium |  |  |  |  |  |
| Zinc | 1.629 (0.862, 3.080) | 1.617 (0.805, 3.249) |  | (-0.455, -0.022) | (-0.317, -0.101) |
| Iron | 0.840 (0.621, 1.138) | 0.796 (0.561, 1.129) |  | (-0.024, 0.185) |  |
| n-3 PUFA |  | 0.083 (0.002, 3.353) |  |  |  |
| Alcohol |  | 0.918 (0.776, 1.085) |  |  | (-0.013, 0.173) |
| Caffeine |  | 1.026 (0.996, 1.058) |  |  | (-0.021, -0.001) |
